# Regulation of the maternal immune system during early bovine pregnancy

**DOI:** 10.1530/RAF-25-0188

**Published:** 2026-05-07

**Authors:** Amber E Thornton, Heloisa M Rutigliano

**Affiliations:** ^1^ Department of Animal, Dairy, and Veterinary Sciences, Utah State University, Logan, Utah, USA; ^2^ Department of Animal and Poultry Sciences, Virginia Polytechnic Institute and State University, Blacksburg, Virginia, USA; ^3^ Department of Veterinary Clinical and Life Sciences, Utah State University, Logan, Utah, USA

**Keywords:** fetal–maternal interface, reproductive immunology, pregnancy, immunological tolerance, embryo

## Abstract

**Abstract:**

A cow’s ability to produce a live calf every year is critical for profitable and sustainable cattle production. Although fertilization rates in cattle are typically high, early embryonic loss remains a major barrier for reproductive success, and immunological rejection of the embryo represents a major contributing factor to this loss. Pregnancy represents an immunological paradox in which the maternal immune system not only tolerates the presence of a semi-allogeneic embryo but creates a uterine environment supportive of its survival and development. Successful pregnancy establishment and maintenance require precise maternal immune system regulation to tolerate embryonic paternal antigens while maintaining protection against pathogens. Disruption of the dynamic balance between pro- and anti-inflammatory responses at the fetal–maternal interface, either through insufficient or through excessive inflammation, can result in pregnancy complications and loss. This literature review examines the immunoregulatory mechanisms in place to create a tightly regulated immunological equilibrium beginning at insemination and continuing through placental development in successful bovine pregnancy, including molecular signaling pathways, leukocyte interactions, and cytokine networks, and discusses the consequences of immunological dysregulation at the fetal–maternal interface. Advancing knowledge in this area holds promise for the development of targeted reproductive management strategies in cattle aimed at minimizing pregnancy loss and improving the efficiency and profitability of cattle production.

**Lay summary:**

A cow’s ability to produce a live calf every year is critical for profitable and sustainable cattle production, but immunological rejection of the embryo, resulting in pregnancy complications or loss, is a significant obstacle. The fundamental role of the immune system is to recognize antigens, or foreign molecules, in the body and induce an inflammatory response against these antigens to protect the host animal. Pregnancy represents an immunological paradox in which the maternal immune system not only tolerates the semi-foreign embryo, which contains paternal antigens, but creates a uterine environment supportive of its survival and development. Immune responses during pregnancy are tightly regulated to balance the need for tolerance of the embryo with the need for protection against pathogens. This literature review explores the regulatory mechanisms used to prevent the maternal immune system from attacking the semi-foreign embryo and describes the consequences of immunological dysregulation during bovine pregnancy.

## Introduction

The ability of a cow to annually conceive, maintain a pregnancy, and deliver a healthy calf is essential for profitable and sustainable cattle production. Despite high fertilization rates, pregnancy loss remains a major limitation, occurring in approximately 40% of early pregnancies in dairy cattle and 48% in beef cattle (Diskin *et al.* 2011, Fair 2015, Reese *et al.* 2020, Albaaj *et al.* 2023). The economic burden of cattle infertility can extrapolate to $700 to $1,100 per head annually, when accounting for cow maintenance costs, production decreases, and lost calf revenue (Prevatt *et al.* 2018). Successful reproduction requires precise maternal immune system regulation to tolerate the semi-allogeneic embryo while maintaining defense against pathogens. Disruptions in immunological balance lead to reproductive failure, reducing herd productivity.

The fundamental role of the immune system is to recognize and respond to antigens by initiating inflammation and activating leukocytes. Pregnancy represents an immunological paradox: the maternal immune system must tolerate paternal antigens while supporting fetal development. Immune responses in the reproductive tract are tightly regulated and must shift from a pro-inflammatory state supporting fertilization and implantation to an anti-inflammatory, tolerogenic state that promotes fetal viability (Samardžija *et al.* 2020). Although cattle-specific studies are limited and pregnancy loss is inherently multifactorial, growing evidence suggests that immunological dysregulation is a significant contributor to bovine reproductive failure (Dunne *et al.* 2000, Rapacz-Leonard *et al.* 2014, Albaaj *et al.* 2023). Tightly controlled immunoregulation is fundamental for sustaining a bovine pregnancy and, thus, for profitable cattle production (Ott 2019, 2020).

## Overview of the uterine immune system

**Figure 1:**
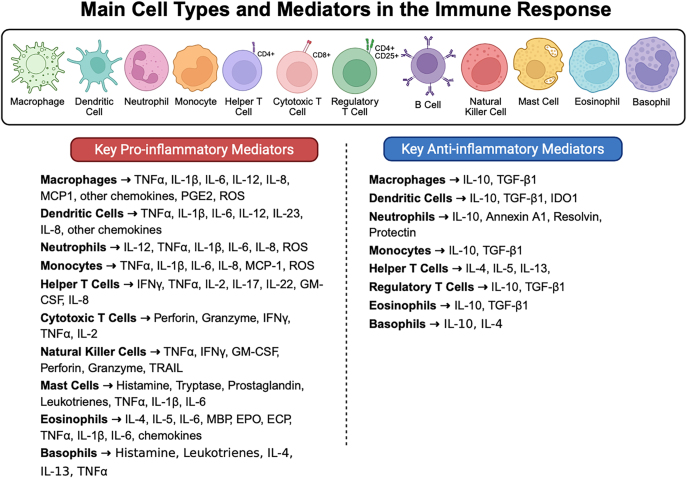
Main immune cell types and their key pro- and anti-inflammatory mediators. The top panel illustrates the major leukocyte populations involved in the immune response, including macrophages, dendritic cells, neutrophils, monocytes, helper T cells, cytotoxic T cells, regulatory T cells, B cells, natural killer cells, mast cells, eosinophils, and basophils. The bottom panels summarize the primary pro-inflammatory (left, red) and anti-inflammatory (right, blue) mediators produced by each cell type. Pro-inflammatory mediators include cytokines (e.g. TNF-α, IL-1β, and IL-6), chemokines (e.g. MCP1 and MCP2), reactive oxygen species (ROS), and other signaling molecules. Anti-inflammatory mediators include IL-10, TGF-β1, IDO1, and specialized pro-resolving mediators. This schematic highlights the dual roles of immune cells in initiating inflammation and promoting resolution and tissue homeostasis. Created with BioRender.com.

The immune system protects the body from microbial invasion through a network of molecules, cells, tissues, and organs that work cooperatively to recognize and respond to antigens. It has complementary parts: innate and adaptive immunity. Innate immunity is non-specific, forms no memory, and acts immediately upon encountering antigens (Marshall *et al.* 2018). It includes neutrophils, macrophages, dendritic cells (DCs), natural killer (NK) cells, mast cells, eosinophils, basophils, and other non-cellular components and is triggered by antigen binding to pattern recognition receptors (PRRs) to initiate inflammation (Hato & Dagher 2015). Key immune cell populations and their associated pro- and anti-inflammatory mediators are summarized in Fig. 1. Antigen-presenting cells (APCs) then process and present antigens on major histocompatibility complex (MHC) proteins to the adaptive immune system, which is characterized by delayed, antigen-specific responses with memory development. Adaptive immune system cells include T and B lymphocytes (Bonilla & Oettgen 2010). Innate and adaptive immunity act jointly to provide both broad immediate protection and pathogen-specific defense.

PRR activation triggers transcription factors, including nuclear factor κB (NF-kB) and IRF3, to stimulate production of pro-inflammatory cytokines. Interleukin (IL)-1β, IL-6, and tumor necrosis factor-alpha (TNF-α) are among the earliest released to induce sickness behavior, increase vascular permeability, and activate and recruit leukocytes (Malazdrewich *et al.* 2001, Rocha *et al.* 2021). Complement proteins, acute phase proteins, antimicrobial molecules, and coagulation factors further enhance pathogen clearance (Mogensen 2009). Chemokine CXCL8 rapidly recruits neutrophils, which defend against pathogens via phagocytosis, degranulation, and antimicrobial nets. NK cells, activated by IL-12, recognize and kill abnormal or infected cells while secreting pro-inflammatory cytokines like interferon gamma (IFN-γ) (Mogensen 2009, Hato & Dagher 2015).

Macrophages are classified into pro-inflammatory M1 and anti-inflammatory M2 subtypes. M1 macrophages defend against pathogens, while M2 macrophages suppress inflammation, promote tissue repair, and stimulate antibody production (Mills & Ley 2014). M2 macrophages phagocytose apoptotic cells and debris, downregulate pro-inflammatory mediators (IL-12, P70, IL-1β, and CXCL8), and upregulate anti-inflammatory mediators (IL-10, IL-6, IL-1Rα, and indoleamine 2,3-dioxygenase 1 (IDO1)). They also function as APCs to clear antigen–antibody immune complexes locally (Yao *et al.* 2019).

Intrauterine DCs are the uterine ‘gatekeepers’ during pregnancy, inducing antigen-specific immunological activation and suppression. While DCs usually modulate uterine immunity, cytokine stimulation permits them to trigger adequate immune activation for protection (Blois *et al.* 2007, Mansouri-Attia *et al.* 2012). DCs secrete pentraxin 3 (PTX3), which mediates pathogen recognition, opsonization, phagocytosis, inflammation, complement system regulation, and autoimmunity (Mansouri-Attia *et al.* 2012, Fair 2015). As the most potent APCs, DCs are required to activate naïve T lymphocytes, influencing their differentiation into Th1, Th2, Th17, and T regulatory (Treg) subpopulations through targeted cytokine secretion. IL-12 and IFN-γ promote pro-inflammatory, cell-mediated Th1 differentiation. IL-4 promotes anti-inflammatory, antibody-mediated Th2 differentiation. IGF-β1, IL-6, and IL-23 promote highly inflammatory neutrophilic Th17 differentiation, and transforming growth factor-β1 (TGF-β1) promotes immunosuppressive Treg differentiation (Saito *et al.* 2010, Hato & Dagher 2015, Huang *et al.* 2020). Th1 and Th17 responses are detrimental to pregnancy, whereas Th2 and Treg responses support it (Mansouri-Attia *et al.* 2012).

Arguably the most influential cytokine in bovine pregnancy is IFN-τ. Type I IFNs, like IFN-τ, have roles in pathogen recognition and response during early gestation. They activate PRR signaling cascades and regulate production of Toll-like receptor (TLR) and pro-inflammatory cytokine mRNAs (Dai *et al.* 2016). Nod-like receptors (NLRs), intracellular PRRs, are activated by type I IFNs and modulate important signaling cascades like NF-kB and IFN pathways (Coutermarsh-Ott *et al.* 2016). Stimulation of PRR and NLR pathways by type I IFNs regulates immune responses through IL and caspase 1 maturation, interferon-stimulated gene (ISG) expression, and interferon regulatory factor (IRF) production (Rocha *et al.* 2021). IFN-τ signaling activates a wide variety of cytokines, chemokines, ISGs, and other inflammatory mediators in bovine pregnancy.

## Overview of bovine pregnancy

Bovine pregnancy involves a series of highly coordinated and appropriately timed events, beginning with oocyte fertilization. During natural breeding in cattle, semen is deposited in the anterior vagina and travels from the vagina, through the cervix, and into the uterus. During artificial insemination, semen is deposited directly into the uterus. Fertilization occurs in the oviductal ampulla, and the zygote remains in the oviduct for three to five days, undergoing mitotic cell divisions and genome activation (Maillo *et al.* 2015). Between days 3 and 5, the zygote forms a morula, an unhatched solid sphere of cells surrounded by the zona pellucida. During this time, it stays in contact with the oviductal epithelium and fluid, which contains carbohydrates, proteins, lipids, and other molecules (Mazzarella *et al.* 2021).

The embryo enters the uterus by day 5 and subsequently develops into a blastocyst between days 6 and 8, in which embryonic cells commit to either the trophectoderm (TE) or inner cell mass (ICM) lineages (Passaro *et al.* 2018, Lonergan *et al.* 2019). The blastocyst receives amino acids, glucose, cytokines, and growth factors via uterine histotroph, the source of nutrition until placental formation (Andrews *et al.* 2025). The blastocyst hatches from the zona pellucida by day 9. The ICM forms a bilaminar disc with two distinct cell layers between days 11 and 12. The outer layer (epiblast) gives rise to the embryo proper, while the inner layer (hypoblast) gives rise to extraembryonic structures (Moreira Da Silva *et al.* 2018, Alberio *et al.* 2021). By day 13, the TE begins rapidly elongating to transition the embryo from a spherical to a filamentous-shaped structure, growing from less than 5 mm on day 14 to 250 mm by day 17 (Curran *et al.* 1986, Moreira Da Silva *et al.* 2018). During this time, the corpus luteum (CL) secretes progesterone (P4), which induces endometrial gene expression changes that promote conceptus development and implantation (Forde *et al.* 2009). A key event during early pregnancy is maternal recognition of pregnancy, which occurs around day 15–17. At this stage, the elongating conceptus secretes IFN-τ, which acts on the endometrium to suppress expression of estrogen receptor α (ESR1) and oxytocin receptor (OXTR), thereby preventing pulsatile prostaglandin F2α (PGF2α) release that would otherwise induce luteolysis. By inhibiting luteolytic signaling, IFN-τ ensures CL maintenance and continued P4 secretion, which are essential for pregnancy survival (Bazer *et al.* 1991, Spencer *et al.* 1996).

**Figure 2:**
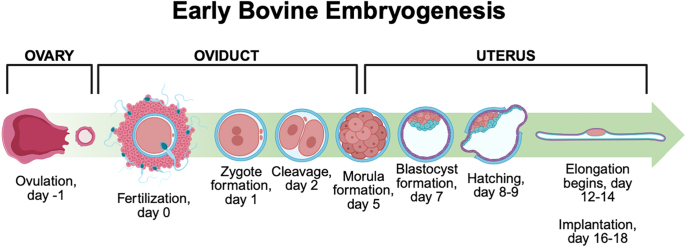
Early bovine embryogenesis. Timeline of bovine embryo development from ovulation through implantation. Ovulation occurs on day −1, followed by fertilization in the ovary on day 0. The zygote forms on day 1 and undergoes cleavage by day 2. Morula formation occurs around day 5, followed by blastocyst formation on day 7. Hatching of the blastocyst occurs on days 8–9. Embryo elongation begins between days 12 and 14, with implantation occurring around days 16–18. Key developmental stages are depicted in the context of their location in the reproductive tract: ovary, oviduct, and uterus. Created with BioRender.com.

Between days 18 and 21, the embryo implants, characterized by superficial interdigitation of the TE microvilli to the endometrium (King *et al.* 1980, Calle *et al.* 2021). An overview of early bovine embryo development from ovulation to implantation is shown in Fig. 2. In the third week of gestation, the embryo undergoes gastrulation, in which a trilaminar disc forms from cell invagination and migration to form three distinct cell layers. The outer layer (ectoderm) forms the nervous system, skin, and hair. The middle layer (mesoderm) forms the muscles, connective tissues, skeleton, blood, blood vessels, kidneys, and reproductive system. The inner layer (endoderm) forms the gastrointestinal and respiratory tracts, liver, endocrine glands, and bladder (Moreira Da Silva *et al.* 2018). The fetal yolk sac, which supplies nutrients to the conceptus, develops by day 23, and chorion and allantois extraembryonic membranes fuse to form the chorioallantois by day 26 (Assis Neto *et al.* 2010, Green *et al.* 2021, Davenport *et al.* 2023). The yolk sac regresses by day 55 when the placenta becomes fully functional (Assis Neto *et al.* 2010). Around day 42, the conceptus forms a fetus with specialized organ systems (DesCôteaux *et al.* 2009). The bovine placenta and placentomes, distinct areas of interwoven fetal and maternal tissues, continue to grow and vascularize until day 190 to support nutrient and waste transfer (Green *et al.* 2021). Bovine gestation lasts 279–287 days, with the most rapid fetal development occurring in the third trimester (Dunne *et al.* 2000).

## Immune response during insemination and fertilization

The first exposure to paternal antigens occurs during insemination, when seminal antigens activate the innate immune system (Akthar *et al.* 2022). Vaginal stimulation during natural breeding increases IFN-γ, IL-17, and TNF-α production, which increases vascular permeability and adhesion molecule expression and recruits neutrophils to the cervix and uterus (Akthar *et al.* 2022, McLean *et al.* 2024). Uterine macrophages, DCs, and mast cells recognize seminal antigens on PRRs, most significantly Toll-like receptors TLR2 and TLR4 (Mogensen 2009, Akthar *et al.* 2022). Seminal antigens then trigger a transient inflammatory response characterized by increased CXCL8, IL-1β, granulocyte-macrophage colony-stimulating factor (GM-CSF), TNF-α, and prostaglandin E synthase (PGES) secretion and TGF-β1 suppression (Elweza *et al.* 2018).

Sperm-endometrium interactions recruit neutrophils, macrophages, and DCs to the uterus. Intrauterine neutrophils are first observed 3 hours post-insemination and completely disappear 10 hours post-insemination (Marey *et al.* 2019). Neutrophils clear the uterus of excess, abnormal, and dead sperm and other pathogens introduced during insemination by phagocytosis, degranulation, and formation of antimicrobial extracellular traps (Hong *et al.* 2017, Akthar *et al.* 2022). Macrophages phagocytose excess sperm and secrete TNF-α, IL-6, and IL-1β to activate endothelial cells, recruit leukocytes, and promote acute phase protein production. IL-6 is required for Th17 differentiation, which recruits neutrophils to the uterus (Schuberth *et al.* 2008, Fair 2015). Intrauterine DCs function as APCs to activate lymphocytes and ultimately establish peripheral tolerance to fetal antigens (Dietl *et al.* 2006, Fair 2015). Semen-induced leukocytes clear the uterus of excess sperm and pathogens and make the uterus receptive to implantation (Talukder *et al.* 2020). These leukocyte responses are strongly influenced by ovarian steroid hormones. 17β-estradiol (E2) and P4 dynamically modulate uterine immune cell populations and activity, coordinating the transition from a pro-inflammatory environment that clears excess sperm and pathogens to an immunosuppressive state that supports sperm survival and embryo tolerance (Marey *et al.* 2016, Cui *et al.* 2022).

Although some sperm are consumed by neutrophils, thousands of sperm reach the oviducts. In contrast to sperm–uterine crosstalk, the sperm–oviduct crosstalk downregulates immune responses to support embryonic survival (Talukder *et al.* 2020). The presence of sperm in the oviduct upregulates TGF-β1 and IL-10 secretion and downregulates TNF-α and IL-1β secretion by oviductal cells (Yousef *et al.* 2016). Bovine oviductal cells also secrete prostaglandin E2 (PGE2), which suppresses neutrophil phagocytic behavior (Marey *et al.* 2013). Thus, the presence of sperm in the bovine oviduct has immunosuppressive effects through neutrophil activity suppression and immunotolerant cytokine secretion to promote sperm survival and fertilization.

**Figure 3:**
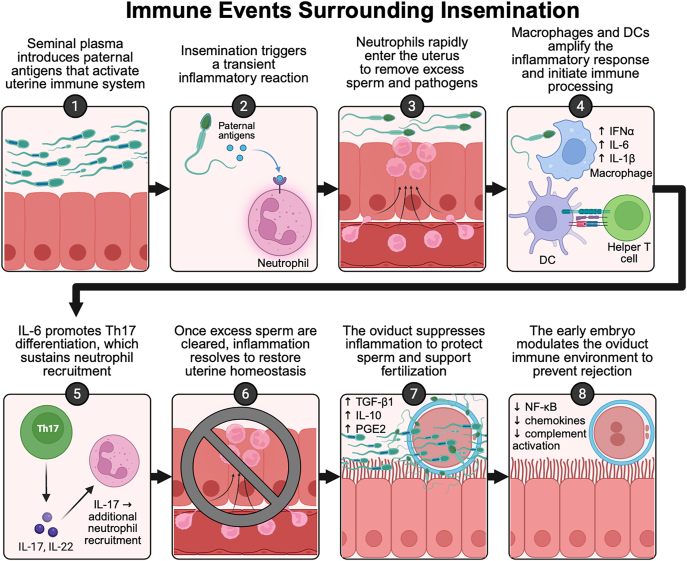
Immune events surrounding insemination in cattle. Seminal plasma introduces paternal antigens that activate the uterine immune system (1). In response, neutrophils detect these antigens and initiate a transient inflammatory reaction (2), rapidly entering the uterus to remove excess sperm and potential pathogens (3). Macrophages and dendritic cells (DCs) amplify the inflammatory response and process antigens, producing cytokines such as IFN-α, IL-6, and IL-1β, which activate helper T cells (4). IL-6 promotes Th17 differentiation, sustaining neutrophil recruitment via IL-17 and IL-22 signaling (5). Once excess sperm are cleared, inflammation resolves to restore uterine homeostasis (6). The oviduct then suppresses inflammation, promoting a tolerogenic environment through TGF-β1, IL-10, and PGE2 to support fertilization (7). Finally, the early embryo modulates the oviductal immune environment to prevent immune-mediated rejection by downregulating NF-κB signaling, chemokine expression, and complement activation (8). This tightly regulated sequence ensures successful sperm clearance, fertilization, and early embryonic survival. Created with BioRender.com.

After fertilization, the embryo’s presence modulates oviductal gene expression to regulate local immune responses, which is crucial to avoid immunological rejection (Rodriguez-Alonso *et al.* 2020). Ovarian steroids, including E2 and P4, adjust oviduct epithelial cell immune activity by suppressing pro-inflammatory signaling and promoting tolerance, which facilitates early embryonic development (Zhu *et al.* 2017, Lonergan & Sánchez 2020). The embryo decreases oviductal inflammation by suppressing NF-kB, chemokines CXCL2 and CCL20, antigen presenters CD74 and TAP-binding protein, and C3 complement protein action (Maillo *et al.* 2015). The major immune events surrounding insemination are summarized in Fig. 3. Despite only remaining in the oviduct for approximately five days, the embryo’s immunoregulatory interactions with oviduct epithelial cells have critical roles in embryonic survival and successful pregnancy establishment in cattle (Talukder *et al.* 2020).

## Immune response during early embryo development and maternal recognition of pregnancy

The morula-stage embryo enters the uterus approximately five days post-fertilization. Unhatched bovine embryos start secreting IFN-τ, an anti-luteolytic immunosuppressive type I IFN, as early as five days post-fertilization (Talukder *et al.* 2017, 2020). IFN-τ’s anti-viral, anti-inflammatory effects are unique to ruminants because it arose after ruminant species had evolved from other mammals (Roberts *et al.* 2003, Sanchez *et al.* 2019). Morula-derived IFN-τ acts locally and systemically to create an immunosuppressive intrauterine environment (Skopets *et al.* 1992). Locally, IFN-τ activates ISGs ISG15, OAS1, and MX2 in uterine epithelial cells that suppress pro-inflammatory transcription factor NF-kB activity and subsequently, TNF-α, IL-1β, and IL-6 production. IFN-τ produced by pre-hatching embryos also upregulates uterine PGES and PGE2 secretion to suppress Th1-type responses *in vitro* (Sajiki *et al.* 2018). Overall, local IFN-τ actions indirectly establish a Th2-type intrauterine environment, suppressing Th1 signaling pathways and upregulating anti-inflammatory, tolerogenic mediators (Passaro *et al.* 2018, Talukder *et al.* 2020). Aside from IFN-τ-mediated signaling, ovarian steroid hormones P4 and E2 critically regulate uterine immune gene expression during this period. These hormones coordinate the timing and magnitude of ISG expression, modulate cytokine production (promoting anti-inflammatory IL-10 and TGF-β1 while downregulating pro-inflammatory TNF-α and IFN-γ), and influence leukocyte recruitment through modulating endometrial expression of adhesion molecule and chemokine transcripts, thereby reinforcing a tolerogenic environment that supports embryo survival and early maternal recognition of pregnancy (Lewis 2003, Forde *et al.* 2011, Magalhães *et al.* 2022, Northrop-Albrecht *et al.* 2022). Systemically, pre-hatching embryo-derived IFN-τ activates ISG15 and OAS1 in peripheral blood mononuclear cells (PBMCs) and neutrophils, increasing immunosuppressive IL-10 expression *in vivo* and pro-inflammatory TNF-α expression *in vitro* to mediate fetal–maternal crosstalk (Shirasuna *et al.* 2012). ISG15 and OAS1 activation, along with PGES production, upregulates TGF-β1 and IL-10 expression by day 7 post-fertilization, suppressing peripheral leukocyte activity *in vivo* (Talukder *et al.* 2018). Peripheral maternal leukocytes respond to signals from the pre-hatching embryo by establishing a Th2-type response during early pregnancy. This suggests that, although maternal recognition of pregnancy is typically considered to occur around day 16 in cattle, ‘very early’ recognition may begin as early as day 8 post-fertilization (Talukder *et al.* 2017).

Around day 7 post-fertilization, the morula develops into a blastocyst. Pre-hatching blastocysts continue secreting IFN-τ and modulate the maternal immune system by altering intrauterine metabolism. The blastocyst’s presence alters uterine lumen fluid composition and influences the expression of endometrial lipoxygenase enzymes. As a result, the blastocyst modulates metabolite concentrations generated by lipoxygenase enzymes, including arachidonic and linoleic acid oxidative metabolism products, which regulate vasodilation, inflammation, and immune function (Sponchiado *et al.* 2019).

During the post-hatching pre-implantation period, elongating conceptuses produce substantially higher quantities of IFN-τ. Because conceptus length is positively correlated with IFN-τ secretion, embryonic elongation is essential to ensure that sufficient IFN-τ is produced for pregnancy progression (Hansen *et al.* 2017). Bovine TE cells secrete IFN-τ in a dose-dependent, autocrine manner to stimulate TE proliferation, which consequently promotes additional IFN-τ secretion (Wang *et al.* 2013). Conceptus-derived IFN-τ prevents luteolysis by downregulating endometrial oxytocin receptors, thereby blocking oxytocin-induced pulsatile PGF2α release, reducing uterine PGF2α-synthesizing enzymes, and increasing luteotrophic PGE2 synthesis (Arosh *et al.* 2016, Talukder *et al.* 2020). IFN-τ stimulates ISG expression, including ISG15, MX1, MX2, OAS1, and RSAD2, in peripheral tissues, such as PBMCs and neutrophils (Hansen *et al.* 2017, Toji *et al.* 2017, Yoshino *et al.* 2023). ISG expression in tissues such as the mesenteric lymph nodes may traffic peripheral leukocytes to the uterus to maintain CL function using IFN-τ as a chemotactic factor (Hansen *et al.* 2017, Rocha *et al.* 2021).

CL preservation is essential for pregnancy viability as it secretes P4, a critical hormone that supports embryo survival and pregnancy maintenance. Immediately after ovulation, the resolution of inflammation and repair of tissue at the ovulatory site are critical for CL formation. Leukocytes, including neutrophils, eosinophils, and macrophages, are recruited to the ovulatory site during ovulation by chemokines (IL-8, CCL2, and CCL5) produced by endothelial cells, fibroblasts, and resident leukocytes (Murdoch & McCormick 1993, Abdulrahman & Fair 2019). Eosinophils and neutrophils contribute to tissue repair and microvasculature reestablishment, while macrophages produce cytokines (TNF-α, IFN-γ, IL-1β, and IL-6), prostaglandins, and angiogenic factors (VEGF and FGF2) that promote angiogenesis, cell survival, and steroidogenesis (Reibiger & Spanel-Borowski 2000, Townson *et al.* 2002, Jiemtaweeboon *et al.* 2011). TNF-α and PGE2 are key regulators of CL vascularization, and luteal-derived P4 supports tissue maintenance by inhibiting apoptosis and modulating local immune activity, including suppression of T lymphocyte proliferation (Turner *et al.* 2011, Abdulrahman & Fair 2019). During early pregnancy, conceptus-derived IFN-τ prevents luteolysis primarily by suppressing endometrial PGF2α release and influencing immune signaling pathways that stabilize the CL (Arosh *et al.* 2016). Leukocytes within the CL continue to produce cytokines and chemokines that sustain luteal cell survival, angiogenesis, and steroidogenesis. Anti-inflammatory cytokines (IL-10 and TGF-β) promote luteal cell viability and limit inflammatory signaling, whereas pro-inflammatory cytokines (TNF-α, IL-1β, and IFN-γ) are associated with structural luteolysis during the nonpregnant cycle (Abdulrahman & Fair 2019, Talukder *et al.* 2020). PGE2, produced by both the conceptus and endometrium under IFN-τ influence, exerts luteoprotective effects by counteracting luteolytic prostaglandins and stimulating P4 synthesis (Arosh *et al.* 2016, Sajiki *et al.* 2022). Intraluteal P4 maintains the CL by inhibiting apoptosis via suppression of Fas and caspase-3 activation (Okuda *et al.* 2004). Through coordinated endocrine and immunological mechanisms, the CL sustains P4 concentrations necessary for pregnancy establishment in cattle.

P4 plays a central role in immune regulation during early pregnancy. Low circulating P4 is associated with reduced pregnancy rates in cattle (Forde *et al.* 2011, Lonergan & Sánchez 2020), whereas elevated P4 supports conceptus development and viability through regulating immune factors such as MCP1, MCP2, PTX3, RSAD2, and TGF-β1 (Forde *et al.* 2009, Mansouri-Attia *et al.* 2012). In ruminants, luteal P4 suppresses both endometrial lymphocyte proliferation *in vivo* and Th1-type IL-1β, IL-6, TNF-α, CXCL8, NOS2, and COX2 production *in vitro*, even in the presence of bacterial antigen (Lewis 2003, Cui *et al.* 2022). Additionally, P4 downregulates uterine eicosanoid synthesis. Eicosanoids, including PGF2α, upregulate immune responses by enhancing pro-inflammatory cytokine production, phagocytosis, and lymphocyte function. By inhibiting eicosanoid production, P4 decreases uterine inflammation and adaptive immune responses (Seals *et al.* 2002). Local immunosuppression by luteal P4 establishes the humoral-mediated Th2 microenvironment crucial for protecting the conceptus from cell-mediated immunological attack. P4 blocks the production of neutrophilic IL-17 by peripheral Th17 lymphocytes. In many species, upregulated IL-17 is associated with pregnancy loss, and its inhibition has been seen to promote embryo viability (AbdulHussain *et al.* 2020, Wang *et al.* 2020, Ma *et al.* 2023). P4 also suppresses production of IFN-γ, TNF-α, and IL-2 and, thus, plays a role in shifting from a cell-mediated Th1 and pro-inflammatory Th17 bias to a humoral-mediated Th2 bias (AbdulHussain *et al.* 2020). Furthermore, P4 promotes Treg proliferation and activity, thereby establishing immunological tolerance to conceptus-derived paternal antigens (Oliveira & Hansen 2008, Tai *et al.* 2008, Mao *et al.* 2010, Maeda *et al.* 2013).

Prostaglandins, namely PGE2, also contribute to immune regulation and are secreted by both the conceptus and endometrium following IFN-τ stimulation. Conceptus-derived prostaglandins act in a paracrine manner to enhance intrauterine ISG activity, including ISG15 (Austin *et al.* 2004). PGE2 is anti-luteolytic and prolongs the lifespan of the CL, thereby sustaining P4 production (Bridi *et al.* 2020). In addition, PGE2 is immunosuppressive and blocks Th1 activity by downregulating T lymphocyte, NK cell, DC, and macrophage function via EP2 and EP4 G-coupled receptors *in vivo*, which initiates a cyclic adenosine monophosphate (cAMP), protein kinase A, and cAMP response element binding protein pathway (Sajiki *et al.* 2022). In bovine PBMCs *in vitro*, PGE2 decreases IFN-γ, IL-2, and INF-α production, blocks Th1 responses, and promotes immunosuppressive M2 macrophages and Treg function (Sajiki *et al.* 2018). Furthermore, PGE2 regulates programmed cell death protein (PD)-1 and lymphocyte activation gene (LAG)-3 activity *in vitro*, which are immunosuppressive mediators that function in a negative-feedback manner to prevent excessive immune responses (Sajiki *et al.* 2018, Meggyes *et al.* 2019). PGE2 also inhibits the proliferation of CD4+ and CD8+ T lymphocytes and upregulates the expression of IL-10, TGF-β1, and signal transducer and activator of transcription (STAT) 3 in PBMCs *in vitro*, thus initiating Th2-type responses and inducing Treg development (Sajiki *et al.* 2018).

Besides its anti-luteolytic effects, conceptus-derived IFN-τ regulates local and systemic maternal immune responses. *In vivo* endometrial exposure to IFN-τ 14 days post-ovulation upregulates ISG15, RSAD2, MCP1, MCP2, PTX3, and IFN-α, which have functions in modulation of innate and adaptive immunity, leukocyte function, and prevention of uterine infection during pre-implantation (Mansouri-Attia *et al.* 2012). Compared to cyclic endometrium, pre-implantation pregnant endometrium has increased expression of ISGs, including IFITM1, TAP1, TAP2, MHC1A, MHC1G, IFIT1, OAS1, OAS2, cytokine-associated genes (CXCL9, CXCL10, CXCL11, FASL, CCL8, IL-15, IL-7, IL-1β, IL-18, and caspase-1), and other immune response-associated genes (IDO, LBP, BPI, PTX3, galectins, and GPB) *in vivo* (Walker *et al.* 2010). These genes have a variety of roles, including immunosuppression, innate immune response, leukocyte recruitment, inflammation, TE adhesion, Treg activity and proliferation, and immunoregulation in the endometrium (Walker *et al.* 2010, Mansouri-Attia *et al.* 2012). Upregulated adaptive immunity and immunosuppressive genes likely induce immune tolerance to the conceptus, while upregulated innate immunity and pro-inflammatory genes likely protect the uterus from infection during local immunosuppression (Walker *et al.* 2010, Mansouri-Attia *et al.* 2012). Conceptus-derived IFN-τ blocks NF-kB and mitogen-activated protein kinase (MAPK) activation to suppress local inflammation and increases MHC class 1 in the embryo, which mediates embryo–maternal interaction and regulates maternal immune responses during the pre-implantation period (Zhu *et al.* 2017). When IFN-τ secretion reaches peak levels around day 19 post-fertilization, the TE implants in the endometrium to form the fetal–maternal interface (Fair 2015).

## Immune response during embryonic implantation

During implantation, regulation of MHC-1 expression in bovine TE is essential for maintaining immunological balance and supporting successful pregnancy establishment. MHC-1 mRNA expression is embryo-stage specific. MHC-1 genes are present from the immature oocyte stage until at least the hatched blastocyst stage, after which global expression sharply declines before selective upregulation in day 14 embryos, suggesting that specific MHC-1 genes contribute to early embryo development (Doyle *et al.* 2009). MHC-1 molecules are classified as classical (MHC-1a) and non-classical (MHC-1b). Classical MHC-1a, which presents antigen to CD8+ cytotoxic T lymphocytes, is largely downregulated in the bovine TE during the first trimester of pregnancy to minimize maternal recognition of paternal antigens (Davies *et al.* 2004). MHC-1a overexpression, as seen in cloned bovine pregnancies, is associated with immune-mediated fetal rejection and pregnancy loss (Rutigliano *et al.* 2016). MHC-1a expression remains low in the TE until gestational month six, when expression gradually increases in the placentomal arcade region until peaking at term to trigger the inflammatory signaling necessary for placental detachment and shedding after parturition (Davies *et al.* 2000, Benedictus *et al.* 2015). Conversely, non-classical MHC-1b, which restricts pro-inflammatory responses by inhibiting NK cells and T lymphocytes, is expressed at a higher concentration in the TE during early pregnancy (dos Santos *et al.* 2015). MHC-1b transcripts are detectable in bovine embryos as early as cleavage *in vitro* and persist in TE tissues throughout gestation (Bainbridge *et al.* 2001, Doyle *et al.* 2009). MHC-1b also accumulates in the bovine endometrium during implantation, likely through soluble molecules released by the TE to promote immunological tolerance to the conceptus (Barreto *et al.* 2025). TE MHC-1 expression is regulated by a variety of conceptus- and endometrium-derived cytokines, including IFN-τ, IFN-γ, IL-4, and leukemia inhibitory factor (LIF) (O’Gorman *et al.* 2010, Al Naib *et al.* 2011). Proper regulation of TE MHC-1 expression during implantation prevents the maternal immune system from mounting an inflammatory or cytotoxic response against the semi-allogeneic conceptus (Davies *et al.* 2004, Doyle *et al.* 2009, Rutigliano *et al.* 2016).

Unlike the predominantly immunosuppressive environment of the pre-implantation period, implantation is characterized by a controlled pro-inflammatory uterine environment. In cattle and other species, implantation is associated with increased uterine production of IL-6, CXCL8, TNF-α, MIP-1β, CX3CL1, and IP-10 (Hannan *et al.* 2006, Van Sinderen *et al.* 2013). Both IL-1β and its receptor are found in the bovine endometrium and embryo by day 8 of pregnancy, and embryonic presence increases intrauterine IL-1β protein (Correia-Álvarez *et al.* 2015), suggesting that pro-inflammatory cytokines contribute to fetal–maternal communication necessary for endometrial receptivity. Cytokines produced at the fetal–maternal interface during implantation attract and activate leukocytes and promote implantation through tissue remodeling and TE migration. Leukocytes, including macrophages and DCs, accumulate abundantly in the uterus during early implantation and secrete both pro- and anti-inflammatory cytokines to induce endometrial remodeling (Nagamantsu & Schust 2010). Uterine NK cells contribute to placentomal vasculature remodeling, although their significance wanes after placental development (Oliveira *et al.* 2013). Leukocyte trafficking and cytokine secretion enhance expression of adhesion molecules in the uterus, facilitating conceptus attachment (Dekel *et al.* 2019).

Macrophages begin rapidly accumulating in the uterus by day 13 post-fertilization and increase in number throughout gestation (Hooshmandabbasi *et al.* 2021). They play a critical role during implantation by orchestrating immunological defense and tissue remodeling, but their functions are highly dependent on subtype and anatomical location, as even closely-associated caruncular and interplacentomal macrophages vary in both morphology and gene expression (Oliveira *et al.* 2010). During implantation, macrophages clear debris, promote angiogenesis, and secrete a variety of pro- and anti-inflammatory cytokines. Their plasticity allows them to shift between pro-inflammatory, Th1-type M1 and anti-inflammatory, tissue repair-associated M2. After implantation, M2 macrophages predominate and are required to sustain maternal immunotolerance. The proportion of M1 to M2 macrophages is important for pregnancy success, as excessive M1 during early and mid-gestation is associated with pregnancy complications and loss (Hooshmandabbasi *et al.* 2021). Like macrophages, uterine DCs help mediate the critical Th1 to Th2 shift and promote local immunosuppression by activating naïve T lymphocytes and inducing Treg responses through cytokine regulation (Saito *et al.* 2010, Wei *et al.* 2021).

During implantation, the maternal immune response shifts from a cell-mediated Th1 bias to an antibody-mediated Th2 bias. Th2 lymphocytes activated by paternal antigen-presenting uterine DCs secrete anti-inflammatory, tissue-protective cytokines, including IL-4, IL-5, IL-10, and IL-13 (Wang *et al.* 2020). Because Th1 and Th17 responses induce cytotoxic and pro-inflammatory responses, their suppression at the fetal–maternal interface is essential for pregnancy maintenance. Cytokines produced by Th1 (IFN-γ, TNF-α, and IL-2) and Th17 (IL-17) cells induce TE cell death, inhibit fetal development, and trigger spontaneous abortions (Chaouat *et al.* 1990). Th2 cells support pregnancy by secreting cytokines that inhibit Th1 and Th17 differentiation, thereby modulating the maternal immune system and promoting M2 macrophage activation via IL-10 (Fair 2015, Mitchell *et al.* 2017, Wang *et al.* 2020). Furthermore, Th2-type cytokines promote B lymphocyte differentiation into antibody-producing plasma cells, generating protective antibodies against paternal antigens for maternal immunotolerance (Maddur & Bayry 2015).

Treg lymphocytes also play a critical role in maintaining maternal tolerance to the semi-allogeneic conceptus. Maternal Treg populations increase significantly in circulation during pregnancy and accumulate at the fetal–maternal interface. Tregs, primed by paternal antigens present in sperm and seminal fluid, induce maternal immunological tolerance by blocking the action of effector helper and cytotoxic T lymphocytes via both cell contact-dependent and contact-independent mechanisms (Saito *et al.* 2010). Exposure to paternal antigens during insemination results in the expansion of antigen-specific Tregs, which create an immunoprivileged environment to protect the conceptus from immunological rejection at the fetal–maternal interface (Clark *et al.* 2008, Huang *et al.* 2020). Intrauterine Treg populations peak shortly after implantation and persist throughout gestation, declining near parturition (Huang *et al.* 2020).

## Immunotolerance mechanisms at the fetal–maternal interface

After implantation, the uterine immune response leans toward a Th2, immunomodulatory state. During the first 38 days, IFN-τ is secreted by TE cells. As in pre-attachment embryos, IFN-τ secretion modulates gene expression in both the uterus and peripheral circulation to induce TGF-β1 and IL-10 by Tregs (Ott 2019). IFN-τ acts in a paracrine manner at the fetal–maternal interface by activating ISGs, including IRF1 and IRF2, that promote uterine receptivity, immunomodulation, and conceptus development (Hansen *et al.* 2017, Rocha *et al.* 2021). IFN-τ also upregulates endometrial monocyte recruiters MCP1 and MCP2 expression *in vivo* (Mansouri-Attia *et al.* 2012). Most macrophages recruited to the fetal–maternal interface are anti-inflammatory M2-type that clear endometrial apoptotic cells to block pro-inflammatory mediator production and induce local immunosuppression (Mansouri-Attia *et al.* 2012, Faas *et al.* 2014).

IDO1 is an immunomodulatory enzyme activated by IFN-τ that inhibits T lymphocyte function through starving endometrial T lymphocytes of the essential amino acid tryptophan by consuming and sequestering it from the uterine microenvironment. Tryptophan deficiency blocks cell cycle progression and induces T lymphocyte apoptosis by causing a mid-G1 phase arrest. Ultimately, T lymphocytes are unable to proliferate in the presence of IDO1 (Lee *et al.* 2002). Besides its T lymphocyte-suppressive functions, IDO1 has been observed to significantly increase IL-4, IL-10, and TGF-β1 production under the influence of IFN-τ (Mohapatra *et al.* 2020). IDO1 suppresses Th1 cells and activates Th2 cells, as increased Th1 cell differentiation in the absence of IL-4 results in pro-inflammatory IFN-γ and TNF-α production, which are associated with pregnancy complications and loss (Mohapatra *et al.* 2020).

Pregnancy-associated glycoproteins (PAGs) are a family of aspartic proteinases expressed by the placenta and are found in high concentrations both at the fetal–maternal interface and in maternal circulation in cattle (Ott *et al.* 2014). PAGs are secreted by trophoblast giant cells (TGCs) of the placenta starting on day 15 post-fertilization, and their concentration rapidly increases. There is a 200-fold increase in PAGs between days 17 and 20 of pregnancy, but they do not reach significant levels until day 24. PAGs are hypothesized to function in immunomodulation and maternal immunotolerance of the conceptus during gestation (Oliveira Filho *et al.* 2020). Dozens of PAG genes are expressed throughout bovine pregnancy, and their individual expression changes as pregnancy progresses (Green *et al.* 2000). Although PAG2 is the most abundantly expressed PAG, other PAGs, including PAG4, PAG6, and PAG11, are expressed in a temporal manner throughout gestation (Telugu *et al.* 2009). Treatment of bovine leukocytes with PAGs *in vitro* reduces the proliferation of granulocytes, monocytes, macrophages, and DCs, suggesting a potential immunomodulatory role (Hoeben *et al.* 1999). PAGs induce CXCL6, a neutrophil chemotactic factor, to be released by bovine endometrial cells from day 24 post-fertilization until the end of gestation (Austin *et al.* 1999, Montgomery 2022). While evidence of PAGs being both immunosuppressive by inhibiting immune cell proliferation and pro-inflammatory by initiating the release of a neutrophil chemoattractant seems contradictory, alpha chemokines like CXCL6 also have other functions besides immunomodulation, including angiogenesis, which is vital for placental development (Balestrieri *et al.* 2008). The functions and relevance of PAGs at the fetal–maternal interface in cattle have yet to be fully described. Still, it is apparent that PAGs play a role in regulating immune responses and inflammation during pregnancy in ruminants.

**Figure 4:**
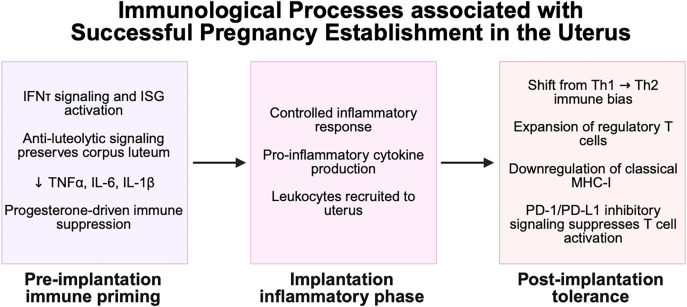
Immunological processes associated with successful pregnancy establishment in the bovine uterus. Pregnancy establishment in cattle can be divided into three immunological phases. In the first phase of pre-implantation immune priming, interferon tau (IFN-τ) signaling is important for maternal recognition of pregnancy and the activation of interferon-stimulated genes (ISGs) that prepare the uterus for receptivity. The corpus luteum is preserved by anti-luteolytic mechanisms, which produces progesterone to maintain the pregnancy and suppress inflammatory responses, including pro-inflammatory cytokines tumor necrosis factor-α (TNF-α), interleukin (IL)-6, and IL-1β. In the second phase of implantation, a controlled inflammatory response increases pro-inflammatory cytokine production and traffics leukocytes to the uterus. This inflammation is essential for the conceptus to impact on the endometrial wall. In the third phase of post-implantation tolerance, the uterus returns to a tolerogenic, immunosuppressive environment through shifting from a Th1 to a Th2 bias, downregulating classical major histocompatibility complex (MHC)-I molecules, recruiting immunomodulatory regulatory T cells, and suppressing T lymphocyte activation through programmed cell death protein and ligand (PD-1/PD-L1) signaling. Created with BioRender.com.

At the fetal–maternal interface, a variety of immune cell types, including monocytes, DCs, effector T lymphocytes, Tregs, and B cells, express programmed cell death protein-1 (PD-1), a transmembrane inhibitory co-stimulatory molecule (Meggyes *et al.* 2019). During pregnancy, PD-1 receptor and its ligand, PD-L1, regulate the balance of T lymphocyte activation and homeostasis, peripheral fetal antigen tolerance, and immune-mediated tissue damage (Sharpe *et al.* 2007). Naïve T cell activation requires two signals from APCs, mainly DCs – presentation of the antigen via an MHC molecule and interaction with co-stimulatory molecules on the DC. The co-stimulatory pathway of T lymphocyte activation involving PD-1 delivers a negative signal that contributes to T cell exhaustion by downregulating T cell-specific cytokine production and inhibiting T cell proliferation and survival (Gianchecchi *et al.* 2013). PD-1/PD-L1 interactions at the fetal–maternal interface also promote the differentiation of naïve T lymphocytes into Tregs and enhance their immunosuppressive activity (Francisco *et al.* 2010). Leukocytes present at the fetal–maternal interface have significantly increased PD-1 receptor expression compared to peripheral leukocytes, likely due to the need for increased immunosuppression at the fetal–maternal interface (Meggyes *et al.* 2019). Furthermore, PD-L1 expression is increased in the endometrial tissue during early bovine pregnancy, correlating with increased P4 concentrations, and its expression is in part regulated by miR-155 and miR-93. miR-155 targets p65, a transcriptional suppressor of PD-L1, and is downregulated in the endometrium of pregnant cattle to preserve PD-L1 expression (Zhang *et al.* 2025). Similarly, miR-93 targets the 3′ untranslated region of PD-L1 to suppress its expression, and the expression of this miRNA is also decreased in the pregnant endometrium. Pregnant cattle that overexpress miR-93 are predisposed to embryo resorption and pregnancy loss (Yang *et al.* 2025). The PD-1/PD-L1 pathway is critical for maternal immunomodulation to regulate leukocyte activity and induce immunological tolerance to the semi-allogeneic conceptus at the fetal–maternal interface (Zhang *et al.* 2015). The immunological processes associated with pregnancy establishment in the uterus, from pre-implantation to post-implantation, are summarized in Fig. 4.

## Consequences of immunological dysregulation in early pregnancy

Inflammation is essential during early pregnancy. During early bovine pregnancy, a controlled, transient inflammatory response is necessary for processes such as implantation and placentation. If the maternal immune system fails to elicit an adequate response, the pregnancy can be lost (Ott 2019). In the absence of pro-inflammatory cytokines, the uterine lining remains unreceptive, leading to implantation failure. Pro-inflammatory cytokines and chemokines are essential for angiogenesis, so their under-expression can lead to abnormal implantation, TE invasion, and, thus, pregnancy failure (Ault-Seay *et al.* 2021). Furthermore, maternal leukocytes, such as macrophages, DCs, and NK cells, are recruited to the uterus during early pregnancy to trigger the low-level, localized inflammation necessary for implantation and placentation (Mansouri-Attia *et al.* 2012). Without leukocyte recruitment, embryos can be lost due to implantation and placentation failure. After the pro-inflammatory peri-implantation stage, the maternal immune system shifts to an anti-inflammatory phenotype.

**Figure 5:**
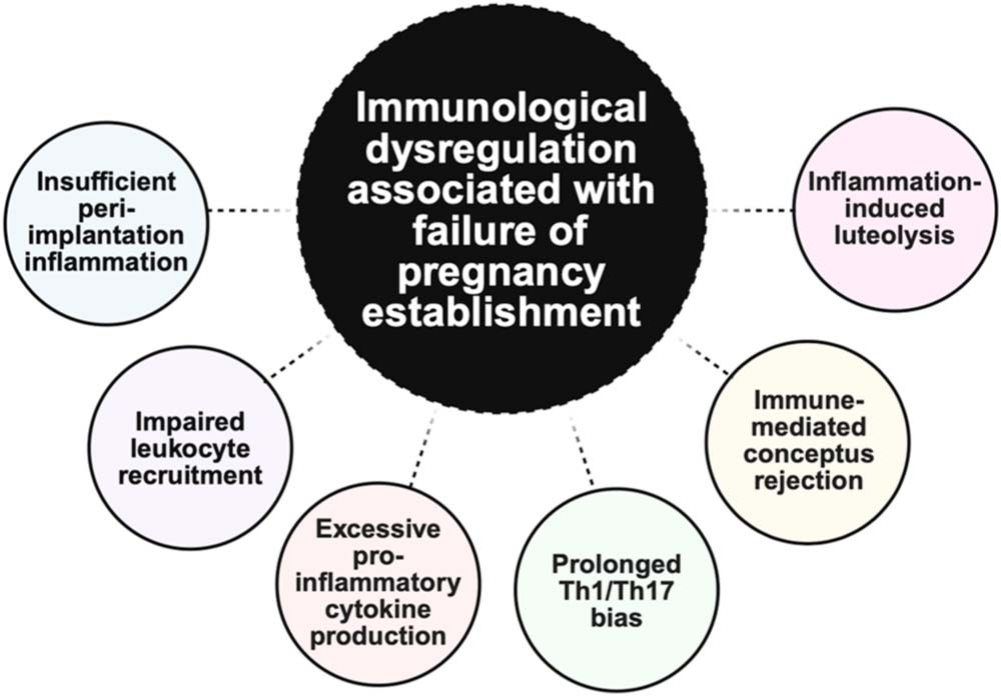
Immunological dysregulation associated with failure of pregnancy establishment. This schematic shows six major causes of pregnancy complications and loss due to immunological dysregulation at the fetal–maternal interface. Key sources of immune-associated pregnancy loss in cattle include insufficient uterine inflammation to allow for successful implantation of the conceptus to the endometrium; impaired leukocyte recruitment to the reproductive tract, disrupting uterine support for embryo; excessive pro-inflammatory cytokine production resulting in excessive endometrial inflammation, vascular damage, and immune rejection of embryo; prolonged Th1/Th17 bias in the uterus, resulting in excessive inflammatory and cell-mediated immune responses; immune mediated conceptus rejection, in which the maternal immune system attacks the semi-allogeneic embryo; and inflammation-induced luteolysis, in which the corpus luteum is lost prematurely and the animal subsequently returns to cyclicity. Created with BioRender.com.

Conversely, excessive inflammation can also severely compromise pregnancy. Elevated levels of pro-inflammatory Th1-type or Th17-type cytokines, such as IL-1β, IL-6, TNF-α, and IFN-γ, induce apoptosis of TE cells, which results in implantation failure and embryo loss (Ott & Gifford 2010). Excessive immunological response and overexpression of pro-inflammatory mediators can also stimulate endometrial PGF2α release, which triggers premature luteolysis, return to cyclicity, and embryo loss (Bazer *et al.* 2010). Furthermore, excessive recruitment of maternal leukocytes, especially cytotoxic T and NK cells, increases the likelihood of immune-mediated conceptus rejection, and persistent excessive uterine inflammation can lead to fetal growth restriction and fetal death (Dunne *et al.* 2000, Sheldon *et al.* 2019). Causes of immunological dysregulation associated with failure of pregnancy establishment in cattle are summarized in Fig. 5.

## Conclusion and future perspectives

Reproductive efficiency is essential for profitable and sustainable cattle production. Pregnancy is an intricate process that requires precise immunoregulation to allow for maternal tolerance of the fetal allograft while maintaining defense against pathogens. The maternal immune system actively balances conceptus recognition with protective responses against infection, using coordinated local and systemic mechanisms to achieve this equilibrium. Disruption of this balance can lead to pregnancy loss, which remains a significant challenge in cattle production. Both over- and under-activation of the maternal immune system represent significant sources of reproductive failure in cattle. Maintaining controlled immunomodulation at the fetal–maternal interface is essential for reproductive success and, thus, sustainable cattle production. Future research should prioritize interspecies comparative and translational studies to identify conserved biological processes and improve cross-species understanding of maternal immunoregulation. Significant advancements have been made to understand the intricate mechanisms involved in maternal immunological tolerance to pregnancy, although most work is still limited to human and mouse studies. Bovine pregnancy is highly comparable to human pregnancy regarding embryo developmental timing and morphology, hormonal control, and immune regulation, and with additional comparative studies, cattle may represent a potential ethically and logistically suitable, highly accessible translational model for both agricultural and biomedical research.

## Declaration of interest

The authors declare that they have no known competing financial or personal interests that could have appeared to influence the work reported in this paper.

## Funding

This research did not receive any specific grant from any funding agency in the public, commercial, or not-for-profit sector.

## Author contribution statement

AT conceived the study and wrote the original draft of the manuscript. HR conceived the study and reviewed and edited the manuscript.
